# National Dental Association

**Published:** 1899-12

**Authors:** 


### Reports of Society Meetings.

### NATIONAL DENTAL ASSOCIATION.

*Second Day.—Morning Session.*

(Continued from page 727.)

THE hour for the special order of business having arrived, Dr. Truman W. Brophy proceeded to exhibit some of his patients who had been operated upon at a very early age for cleft palate and hairlip.

One, a girl, now ten years old, had been operated upon at the age of ten days, having had a double hairlip with a wide cleft of both hard and soft palate. The fissures of the lip extended into the nostrils, the intermaxillary bones and the central portion of the lip being rudimentary.

Another patient, a babe of three months old, had been operated upon at the age of three weeks for single hairlip and cleft palate, a brother of the first patient, four of six children born of the parents being afflicted.

Of the six children, the oldest was entirely free from deformity; the second was the girl first described; the third had a harelip; the fourth a double hairlip and cleft palate; the fifth child was normal; the sixth, the babe presented as described. The paternal grandfather was similarly affected, confirming the opinion that nearly all such cases are of hereditary origin.

In connection with the presentation of these patients Dr. Brophy read a paper entitled "The Radical Cure of Congenital Cleft Palate," of which an abstract follows:

While it is true that the methods pursued by Dr. Brophy are not generally practised, and have been severely criticised by those who do not fully comprehend them, it is also true that many of the most distinguished surgeons who formerly questioned these methods are now their most enthusiastic advocates. Dr. Brophy said that his first cases were undertaken with a great deal of hesitation, as it was a transgression of all the long-accepted rules of surgical procedure. But the question of early operation has passed the experimental stage. The results in hundreds of operations performed at from ten days to three months of age have fully justified the practice. The reasons for operating as early as practicable after birth are as follows:

*First.* The surgical shock is less, as children react better; all mental apprehension is eliminated, alarm and dread being among the most powerful factors in producing shock; the nervous system is not well developed in the infant and they are not capable of receiving the impressions that they would later in life.

*Second.* Before the bones are fully calcified they may be bent or moved without full fracture, and hence the injury is really less than after more complete calcification.

*Third.* If the muscles are very early brought into action, they develop instead of atrophy, and hence a good velum is secured, with plenty of tissue. Later in life, after the parts have shrunken through non-use, they can rarely be made to subserve the same purpose as when developed through natural employment.

It was predicted by eminent surgeons that when, as the result of Dr. Brophy's operation, the upper jaw is reduced in breadth (in the first case shown to about three-quarters of an inch, the lower jaw having twice that measurement), it would always remain contracted, and the superior teeth erupt, if at all, considerably within the inferior arch.

But even had this been the case, it would have been within the province of orthodontial methods to correct it; but it has invariably been the case that the arch has spread and the teeth at eruption have assumed nearly or quite their usual relations. All the tissues, bony as well as soft, develop naturally, according to accepted types.

Another great advantage is that the operation is performed before faulty habits of enunciation have been acquired, and normal speech is assured. An essential point is that the operation upon the palate should be made before operating upon the lip, thus allowing free access, as all the space that can be secured is required. The lip operation can be performed at any time. For the operation the edges of the fissure should be thoroughly removed; merely scraping the mucous membrane is not sufficient. Trim the opposing edges of the bones as well, to secure sufficient exudate, so essential to a perfect union. The soft bones of the hard palate and alveolar process of young patients are easily cut through with the knife.

It may be necessary, in order to close the fissure, to divide the mucous membrane and bone through the malar process, dividing a maximum amount of bone and a minimum amount of membrane, the bone being then readily moved towards the median line and the cleft closed by approximation of the two sides. After the sutures are inserted (a braided silk suture followed by silver wire No. 19, the ends protruding through eyelets in lead plates fitting the convexity of the buccal surface, and twisted together), if the parts are kept antiseptically clean, they will unite kindly and the palate be restored so that its full function may be performed. The germs of some of the teeth may possibly be destroyed, or some of them be imperfectly developed, but operative dentistry is competent to remedy this. The alveolar process developing with the teeth is a pronounced factor in the formation of the jaw, guiding the teeth into proper position.

DISCUSSION.

In the discussion of this paper

*Dr. W. C. Barrett* said that if dentistry had never given anything to the world but this single operation for the radical cure of cleft palate, dentistry would be immortal. It is an operation that stands on its merits and claims the admiration of the world. There once stood a Man of whom it was said that He anointed the eyes of the blind and they saw; that He touched the lips of the dumb and they spoke. The days of miracles are not past. Through the Brophy operation those who were destined to dumb solitude are given back to the world with the oral functions restored. This operation is the very climax of all operations in the cases to which it is adapted. Of over five hundred cases, every one, so far as known, has been a success; only one child has died subsequent to the operation, and that not because of it. I knew of the first operation when it was made. I then said the tissues will unite, but there will be a bad condition of things afterwards. If a vault of one and one-half inches breadth is reduced to three-quarters of an inch, what will be the position of the teeth? They will be away inside of the inferior arch. I was astonished when Dr. Brophy pointed out to me that the deciduous teeth were erupting normally and in almost complete occlusion. I do not know by what mysterious operation nature has brought about this coaptation. It has taken on the type of normality, and the child speaks plainly.

*Dr. M. H. Cryer* said that he does not believe in *miracles*, past, present, or future, but he believes in the ingenuity of man in devising ways of remedying the defects of nature. At an appropriate time he said he would demonstrate why the jaw is not narrowed by this operation. The upper jaw is normally smaller in infancy and old age; the spreading is done by the bone which carries the teeth.

The regular order of business was now resumed, and the work of Section III. again taken up. Dr. J. N. Crouse read a paper entitled "A Résumé of the Important Changes that have taken place in Dentistry during the last Thirty-five Years."

In this paper Dr. Crouse described the facilities (or rather the lack of facilities) for the study of dentistry when he began the study, with only four dental colleges in the world, with a course of study of two years of four months each; before the introduction of the rubber dam, of cohesive gold, or of the mallet, or the invention of the dental engine. He described the Arthur system for the prevention of decay, and showed *per contra* the importance of the restoration of full contour, although shallow cavities on the proximal surfaces of the six anterior teeth may often be advantageously obliterated by judicious cutting from the proximo-lingual surface, when, in the majority of cases decay will not recur. Dr. Crouse briefly reviewed the work of Dr. W. D. Miller in the discovery of the real causes of dental decay; also the "new departure" theories promulgated by Dr. Foster Flagg and others, and the injury done in carrying out these theories. The conclusions from this review of the past are,

*First.* That the practice of filling with non-cohesive gold in the form of cylinders is too valuable to be abandoned, the use of cylinders being specially indicated at the cervical margins of large proximal cavities, finishing and contouring with cohesive gold.

*Second.* That heavy gold can be packed in a cavity more uniformly and with less pressure than lighter gold.

*Third.* That when the ravages of decay are the most active, and the walls very frail, gold is the most valuable material to use.

*Fourth.* That for very many cases gold is not so desirable, not because the teeth are too soft, but because the peridental membrane is likely to be disturbed and injured.

*Fifth.* That it is wise practice to protect the danger-points by extension of the cavity where decay is likely to recur beyond the filling.

*Sixth.* That the occluding surface is impaired by open spaces; this should be overcome by contouring.

*Seventh.* That the great question yet unsolved is to ascertain the cause of dental caries; what conditions cause the radical difference in different mouths, or in the same mouth at different times.

DISCUSSION.

*Dr. S. B. Palmer*, being called upon to open the discussion of this paper, said that he would only speak to that portion touching upon the cause of dental caries. He had been represented as holding a theory opposed to the views of Drs. Miller, Black, and Williams, and wished to correct this misrepresentation. Also in regard to the use of gold in cavities in teeth with undeveloped dentine, dentine which is yet receiving support from the pulp; in the presence of gold the conditions are changed, become abnormal, and the dentine ceases to calcify in contact with a filling which is a non-conducting medium. Gold does not have that effect in a tooth of mature normal structure, but in teeth of a low grade, with highly organized dentine, calcification is checked, calcic deposits cease, and decay continues around the filling. Under proper conditions gold preserves the tooth, but not in the conditions mentioned.

*Dr. G. V. Black* did not hear the paper, but had heard some of the discussion. It is very interesting to look back and note the development of thought in the line of operative dentistry. In conservative dentistry the operation of filling teeth holds the principal place. The study of dental caries, and of operations for the arrest or limitation of the injuries produced by caries, is a most interesting subject. The older men among us began their work during the reign of soft gold, or, more correctly speaking, non-cohesive gold, the successful use of which required that the cavity have four good walls, cylinders being the only proper form in which to use non-cohesive gold. Then came the use of cohesive gold, and the heavy foils 60, 120, and even double that, and back again, from 11 to 20 being now considered a heavy foil. Then came the crystal gold, the precipitated gold, that is so beautiful and works so nicely; and yet there are conditions, as may be demonstrated, that will forever prevent this beautifully working material from being *the gold* for our use. The welding property of gold should be studied thoroughly by every student in our schools. Chemical experiments should be made and our younger men given a better understanding of the properties of gold. There is much to be found in the old books which is being lost in the present generation, which is well worthy of their careful study. Unless we connect the present with the past we cannot judge what are the waste products and what still holds good. The present is but the past made more perfect.

*Dr. J. Y. Crawford.*—Time does not permit of comment upon all that is called up by the paper read by Dr. Crouse. It is right that we should recall the history of dental operations, of the earlier dental surgical operations. It was said last night that the study of dental history belonged to the last year of the college course, and yet we require that the student shall be familiar with the history of his country before he enters the dental college. How much more should this be the case with the history of his chosen profession. He should take it up at his entrance into the college, that he may have some conception of the wonderful field that lies before him. The last proposition in the paper enunciates the true philosophic doctrine in the study of the etiological factors in caries. This disease, which has the elements of infection and contagion, will yet yield to the great law of immunity. We know there is a condition of partial immunity, and if partial immunity be possible, perpetual immunity should also be a possibility. I see no reason why caries should not be subject to the same law of immunity as small-pox.

*Dr. C. Edmund Kells* desired to take exception to the view taken by Dr. Crouse of the "new departure" theories. Much has been said about the bulging and spheroiding of amalgam, but it will be found, if carefully examined, that the cavities have not been properly prepared at the margins; the enamel edges have broken off and left the amalgam standing; hence the appearance of bulging out of the cavity.

*Dr. H. J. McKellops.*—I am not an amalgam-stuffer, as you all know, but if you will use amalgam, after you have cleaned your cavity out nicely put in a good foundation of oxyphosphate cement, and you will have no decay under it. I ask you to investigate that point; try an oxyphosphate lining for cavities in children's teeth until they are strong enough for gold. I defy you to produce a case of decay under oxyphosphate if it has been properly put in. I have been practising dentistry nearly sixty years, and I have never become used to amalgam nor found any necessity for it, and yet you continue to place that stuff into teeth; it wears out, and you have to replace it; it does not save the teeth. There are other materials that do protect the teeth. Why do you use it?

*Dr. Noble.*—In regard to the "Arthur" method, I have a little incident in mind that bears on that point. In 1854 a young man came to me to have a filling inserted in the superior left lateral incisor. I found that it was a very shallow cavity, and that a very little cutting from the lingual surface would entirely remove it. I could see no objection to doing that, so I cut a very little away and polished the surface and dismissed him, satisfied that I had done what was right. But the young man's father came to me, very much enraged, saying that I had ruined his son's tooth, and threatened to have me sued for damages. The case was examined by Dr. Maynard and others, and some of them thought that I had done wrong in cutting away the tooth. That was in 1854, and I saw that man only a few weeks ago, and that tooth is perfect to-day. Other teeth that were filled at that time are badly broken down, and if I had done for them what I did for the lateral incisor, it would probably have been better for them. I fully believe that for shallow proximal cavities in the incisor teeth a little cutting away from the lingual surface is better than to dig a big hole in the tooth.

*Dr. W. C. Barrett.*—As we get old and lazy we grow fonder of the cements. Dr. McKellops is neither old nor lazy; he is immune to the defects to which some of us plead guilty. In my younger days nothing but gold could satisfy me. But I am getting old and lazy, perhaps, and I find that I can satisfy both my conscience and my patients with other materials than gold, and I believe that I have done my duty so long as I preserve the integrity of the teeth. I admit that I cannot comprehend the mysterious nature of amalgam. I have followed the work of Dr. Black. I have investigated the theories of Dr. Palmer, though I cannot say that I agree with him that tooth substance can be dissolved through the agency of electrolysis; yet caries goes on around the work of the best of us under certain circumstances. At the bottom of it all gold is the only thing that really satisfies the longings of the inmost soul of the honest dentist. But there are cases when we must use the next best thing, and that is one of the plastics, renewed again and again. There may be a little loss of tooth-substance, but it never need approach the pulp; and as we get older and lazier we find that we rely more and more upon it. It now satisfies my conscience, and I really believe I render my patients better service and greater satisfaction with oxyphosphate renewed as necessary than I could with gold.

*Dr. Crouse*, in closing the discussion, said that he had two or three objects in view in this review of the past, but the discussion of cements was not one of them; that will come up later in connection with other papers. He wished to emphasize his first proposition. "Cylinder-filling" is almost a lost art, but individually he would not like to practise dentistry if he had to give up cylinders. If a large cylinder is laid at the cervical margin of a cavity and cohesive gold driven into that, a more accurate operation will result than if all cohesive gold is used. He desired to make a special plea for the use of gold in difficult cases. Where we have frail walls, there we want a cohesive gold. There is nothing like gold to keep the profession up to the standard as skilled operators. He said, "I am an enthusiast for gold; when I get it just right, perfect, I am glad I am a dentist. If I ever use plastics it is when I get lazy. As to recurring decay the filling must be carried beyond where decay has gone. Remove all defective structure, and be sure you go far enough."

*Dr. G. V. Black.*—I have had an experience now of five years with an amalgam that does not flow, that neither shrinks nor expands. I have made fillings under conditions such that I could observe them closely, and I have seen neither shrinkage nor expansion, and not a single discolored margin so far as I am able to judge, and I am watching them closely. It is now my opinion that fillings made of an amalgam that is properly constituted and annealed, will give this good result.

*Dr. Crouse.*—One word more in closing. I omitted to speak of the method of removing superficial decay by cutting it away early. I recently removed a lateral and a central incisor from which, twenty-eight years ago, I filled one side and cut away the other. I have since had to refill the filled cavities, but the side that was cut away has never decayed. In all my experience there has not been, to my knowledge, a recurrence of decay when it was early removed by cutting away and polishing the surface. It does not come back.

On motion, adjourned to 7.30 P.M.

(To be continued.)

